# The Organ Trail: A Review of Biomarkers of Organ Failure

**DOI:** 10.3389/fonc.2020.579219

**Published:** 2020-11-11

**Authors:** Long Dao, Dristhi Ragoonanan, Sofia Yi, Rita Swinford, Demetrios Petropoulos, Kris M. Mahadeo, Shulin Li

**Affiliations:** ^1^ Department of Pediatrics, The University of Texas MD Anderson Cancer Center, Houston, TX, United States; ^2^ Division of Pediatric Nephrology, University of Texas Health Science Center Houston, Houston, TX, United States

**Keywords:** oncology, organ failure, liquid biopsy, biomarkers, diagnostics

## Abstract

Pediatric organ failure and transplant populations face significant risks of morbidity and mortality. The risk of organ failure itself may be disproportionately higher among pediatric oncology patients, as cancer may originate within and/or metastasize to organs and adversely affect their function. Additionally, cancer directed therapies are frequently toxic to organs and may contribute to failure. Recent reports suggest that nearly half of providers find it difficult to provide prognostic information regarding organ failure due to unknown disease trajectories. Unfortunately, there is a lack of uniform methodology in detecting the early symptoms of organ failure, which may delay diagnosis, initiation of treatment and hinder prognostic planning. There remains a wide array of outstanding scientific questions regarding organ failure in pediatrics but emerging data may change the landscape of prognostication. Liquid biopsy, in which disease biomarkers are detected in bodily fluids, offers a noninvasive alternative to tissue biopsy and may improve prompt detection of organ failure and prognostication. Here, we review potential liquid biopsy biomarkers for organ failure, which may be particularly useful among pediatric oncology patients. We synthesized information from publications obtained on PubMed, Google Scholar, clinicaltrials.gov, and Web of Science and categorized our findings based on the type of biomarker used to detect organ failure. We highlight the advantages and disadvantages specific to each type of organ failure biomarker. While much work needs to be done to advance this field and validate its applicability to pediatric cancer patients facing critical care complications, herein, we highlight promising areas for future discovery.

## Introduction

Remarkable therapeutic advancement in pediatric oncology may be limited by long-term toxicity, and in particular, acute and/or chronic organ dysfunction/failure (1, 2). The most common reason for intensive care unit (ICU) admission in pediatric cancer patients is acute organ failure (3). While the definition of multiple organ dysfunction syndrome in pediatric patients (p-MODS) remains to be sufficiently determined, there is growing recognition that decrements in organ function may reliably predict stepwise increases in mortality rate and current binary descriptors of normal function versus dysfunction may hinder opportunities for earlier intervention (4, 5).

Among critically-ill pediatric oncology patients, overall ICU mortality ranges from 12-15 % in those with solid and hematologic malignancy, and can be as high as 60% in stem cell transplant (SCT) recipients (6, 7). Organ failure may occur acutely, sub-acutely or progress to chronic disease, and numerous studies have shown early detection may lead to critical timely intervention and improved outcomes. Currently, the gold standard diagnostic criteria for organ failure are organ specific and are based on laboratory, clinical or a combination of both parameters as summarized in **Table 1** (8–15). Although these tests are widely used to diagnose organ failure, they are not predictive. Multiple scoring systems have been developed to attempt to predict the likelihood of developing single as well as multi-organ failure (**Table 1**). However, these are based on clinical evidence when organ dysfunction is already present.

**Table 1:** Diagnostic Criteria for Organ Failure (8–15).

Organ	Laboratory Test	Clinical Parameter	Grading System	Prognostic Scoring System
Heart (8, 9)	NT-pro BNP	Exercise Tolerance	NYHA-functional classification based on clinical parameters	
Lung	paO_2_ paCO_2_		None	
Liver (10, 11)	ALT AST			MELD(based on bilirubin, creatinine, INR) PELD(based on bilirubin, albumin, INR)
Kidney (12)	Creatinine	Urine output	KDIGO	
Multiorgan (13–15)	Creatinine Bilirubin Platelet count paO_2_	GCS Mean arterial pressure Need for mechanical ventilation FiO_2_		SOFA pSOFA APACHE

NYHA, New York Heart Association; paO_2_, arterial partial pressure of oxygen; paCO_2_, arterial partial pressure of carbon dioxide; ALT, alaninine aminotransferase; AST, aspartate aminotransferase (changed order); MELD, model for end-stage liver disease; PELD, pediatric model for end-stage liver disease; KDIGO, Kidney Disease Improving Global Outcomes; GCS, Glasgow Coma Scale; SOFA, Sequential Organ Failure Assessment; pSOFA, Pediatric Sequential Organ Failure Assessment; APACHE, Acute Physiologic Assessment and Chronic Health Evaluation.

In this review, we evaluate currently available biomarkers, their diagnostic accuracy, clinical applicability and potential impact on the prediction and early detection of organ failure. The role of chemokine and cytokine expression in the progression of organ failure has been more extensively characterized and are not included in this review (16–19). Here, we review various noninvasive biomarkers including microRNA, cell free DNA, histones, exosomes, circulating mitochondria and circulating endothelial cells as promising potential biomarkers of organ failure (**Table 2**).

**Table 2:** Summary of Studies Examining Organ Failure.

Organ	Author (year)	Setting	Biomarker	Gold standard control used in the study	Sample size and Study Design	Result	Clinical significance
**Heart**	Cakmak et al. (20) 2015	Chronic congestive heart failure	miRNA-182	NT-pro BNP	Prospective Study Age: ≥ 18 years n=42 HF patients grade II, III, and IV n = 15 Healthy controls	AUC NT-pro BNP :0.35 miRNA- 182: AUC 0.695	miRNA-182 is a potential prognostic marker for systolic heart failure
**Lung**	Zhu et al. (21) 2017	ARDS	miRNA-181a miRNA-92a miRNA-424	LIPS	Case control Study Age: ≥ 18 years n= 78 Patients with ARDS n= 78 Critically ill matched controls	AUC LIPS: 0.708 LIPS + miRNA-181a + miRNA-92a + miRNA-424: 0.723	miRNA profiling combined with the LIPS score can improve the risk estimate for ARDS
	Njock et al. (22) 2019	IPF	miRNA in Sputum exosomes	DLCO/VA	Prospective Study Age: ≥ 18 years n= 16 patients with IPF n= 14 healthy subjects	miRNA-33-a-5p + Let-7d-5p + miR-142-3p AUC: 0.978 Sensitivity: 93.75% Specificity :80%	This combination of miRNAs is a potential biomarker for severity of lung disease in IPF
	Guiot et al. (23) 2019	IPF	Nucleosomes	DLCO/VA	Prospective Study Age: ≥ 18 years n= 23 patients with IPF n= 27 patients with IPF treated with antifibrotic therapy n=27 healthy volunteers	Nucleosomes AUC: 0.93 Sensitivity: 91% Specificity: 80%	Nucleosomes in patients with IPF are a potential diagnostic as well as treatment response biomarker
**Liver**	Tao et al. (24) 2019	Liver dysfunction in patients with chronic hepatitis B	miRNA-125b-5p miRNA-122	MELD score	Prospective Study Age: ≥ 18 years n=136 ACLF n=90 moderate to severe liver damage n= 100 normal hepatic function	AUC MELD score : 0.799 miRNA-125b-5p + miRNA-122 : 0.898	Combined miRNA-125b-5p and miRNA-122 was a superior predictor of outcome of ACLF in patients with Hepatitis B
	Zheng et al. (25) 2016	HBV related ACLF	miRNA-130a	MELD score	Prospective study Age: ≥ 18 years n=39 patients with ACLF n=20 healthy controls	AUC MELD score: 0.86 miRNA-130: 0.74	miRNA-130a alone is not superior to the MELD score in predicting outcomes in liver failure
	Schutz et al. (26) 2017	Liver transplant	cfDNA	AST	Prospective Study Age : ≥ 18 years n= 107 patients post liver transplant with no evidence of graft rejection n= 17 patients posttransplant with acute liver rejection	AST AUC: 0.957 Sensitivity: 82.1% Specificity: 95.7% cfDNA AUC: 0.971 Sensitivity: 89.3% Specificity: 95.7%	CfDNA allowed for earlier and more sensitive discrimination of acute rejection in patients post liver transplant as compared to conventional LFTs
	Lambrecht et al. (27) 2015	Liver fibrosis in patients with HBV or HCV	miRNA	Fibroscan	Prospective Study Age: ≥ 18 years n= 39 patients with early stage liver fibrosis with HBV or HCV n= 14 healthy subjects	miRNA in exosomes HBV: 0.8421-0.9802 HCV:AUC: 0.8745-0.9841	MiRNA in combination with fibroscan techniques is can potentially discriminate between stages of liver fibrosis
**Kidney**	Merkle et al. (28)	AKI post cardiac surgery	cfDNA	Creatinine	Prospective Study Age: ≥ 18 years n= 21 patients post cardiac surgery with AKI n= 37 patients post cardiac surgery without AKI	Creatinine AUC: 0.688 Sensitivity: 87.5% Specificity: 56.8% cfDNA AUC: 0.804 Sensitivity: 87.5% Specificity: 64.9 %	CfDNA is a valuable potential predictor of AKI post cardiac surgery
	Aguado-Fraile et al. (29) 2015	AKI	miRNA-26b-5p miRNA-27a-3p miRNA-93-3p miRNA-127-3p	Cystatin C*	Prospective Study Age: ≥ 18 years n= 35 ICU patients with AKI n= 41 patients undergoing cardiac surgery n = 20 healthy controls	AUC Cystatin C: 0.73-0.76 miRNA-26b-5p: 0.908 miRNA-27a-3p: 0.888 miRNA-93-3p: 0.887 miRNA-127-3p:.863	The combination of miRNA-26b-5p, miRNA-27a-3p miRNA-93-3p and miRNA-127-3p is superior and earlier predictor of AKI than serum creatinine in patients undergoing cardiac surgery
	Fan et al. (30) 2019	AKI post MI	miRNA-24 miRNA-23a miRNA-145	Serum NGAL	Prospective Study Age: ≥ 18 years n= 108 patients admitted to the CCU	AUC NGAL: 0.735 miRNA-24 + miRNA-23a+ miRNA-145: 0853	The combination of miRNA-24 + miRNA-23a+ miRNA-145 is a potential predictor of AKI post AMI that is superior to serum NGAL
	Sole et al. (31) 2017	Chronic kidney disease	Urinary exosomes	Healthy patients	Prospective Study Age: ≥ 18 years n= 32 patients with lupus nephritis n= 15 patients with non-lupus chronic kidney disease n= 20 healthy controls	Urinary exosomes AUC: 0.946 Sensitivity: 94 % Specificity :82%	Urinary exosomes are a potential biomarker for detecting renal fibrosis
	Hu et al. (32) 2018	AKI	Urinary mtDNA	Creatinine	Prospective Study Age: ≥ 18 years n= 125 patients admitted to the Surgical ICU	Urinary mtDNA AUC: 0.814-0.821 Serum Creatinine AUC: 0.724	Urinary mtDNA was superior than serum creatinine in predicting AKI
**Pancreas**	Ha et al. (33)	Pancreatitis	EPC	CRP	Prospective Study Ages ≥ 18 years n= 30 patients with mild acute pancreatitis n= 30 patients with severe acute pancreatitis n= 20 health volunteers	CRP: AUC: 0.86 Sensitivity: 73.3% Specificity : 96.7% Endothelial progenitor cells AUC: 0.926 Sensitivity: 90% Specificity : 83.3%	EPCs are a potential predictor of severe acute pancreatitis
	Liu et al. (34) 2018	Persistent organ failure in patients with severe acute pancreatitis	Histones	APACHE II criteria	Prospective Study Age: ≥ 18 years n = 236 patients with acute pancreatitis n =47 healthy controls	APACHE II criteria AUC: 0.74 Endothelial progenitor cells AUC: 0.92	Circulating histones are a potential biomarker for predicting persistent organ failure in patients with acute pancreatitis
**Multi -Organ failure**	Tapia et al. (35) 2019	Survival outcomes in patients with sepsis	EPC	SOFA score	Prospective Study Age: ≥ 18 years n = 43 ICU patients n= 27 ICU patients with septic shock n= 16 Critically ill patients without septic shock(16) n=22 healthy volunteers	AUROC SOFA score: 0.37 EPC: 0.73	EPCs can be used as biomarkers for predicting survival outcome sin patients with sepsis

*Historical control as per previously reported studies.

miRNA, micro ribonucleic acid; NT-Pro BNP, B-type natriuretic peptide; AUC, area under the curve; ARDS, acute respiratory distress syndrome; PaO_2_/FiO_2_, ratio of the arterial pressure of arterial oxygen to fractional inspired oxygen; LIPS, Lung Injury Prediction Score; IPF, Idiopathic Pulmonary Fibrosis; DLCO/VA, diffusing capacity of the lungs for carbon monoxide/alveolar volume; MELD, Model for End Stage Liver Disease; ACLF, acute on chronic liver failure; cfDNS, cell free deoxyribonucleic acid; AST, aspartate aminotransferase; LFT, liver function test; HBV, hepatitis B Virus; HCV, hepatitis C virus; AUROC, area under the receiver operating characteristic curve, AST: Aspartate aminotransferase; HCV, Hepatitis C Virus; AKI, Acute Kidney Injury; NGAL, neutrophil gelatinase-associated lipocalin; CCU, coronary care unit; ICU, intensive care unit; AMI, acute myocardial infarction; CRP, C-reactive protein; mtDNA, mitochondrial deoxyribonucleic acid; APACHE, Acute Physiology and Chronic Health Evaluation; SOFA, Sequential Organ Failure Assessment Score.

## Cell Free DNA

Cell-free DNA are small degraded fragments of DNA, derived from the apoptosis of nucleated cells and circulate freely in the blood plasma (36). Under normal physiological conditions, healthy subjects have lower levels of cfDNA compared to patients with systemic illnesses, making them a possible biomarker of organ failure (37).

CfDNA has been studied in the blood of patients undergoing cardiac surgery to predict late acute kidney injury (AKI). The ROC generated from cfDNA of patients had improved detection of AKI (AUC = 0.804) compared to the ROC generated from the serum creatinine of patients (AUC = 0.688) (28). Karlas et al used total plasma cfDNA to assess hepatic fibrosis in comparison to current methods such as transient elastography. Plasma cfDNA concentrations did indeed correlate with degree of hepatic fibrosis and severity in non-alcoholic fatty liver disease (p value < 0.001) (38).

While the above studies utilize total plasma cfDNA, a recent study has shown that in the plasma of lung transplant patients, donor-derived cfDNA (ddcfDNA) could be identified and quantified, with patients with increased levels of ddcfDNA having a higher risk of allograft failure (39). Thus, ddcfDNA can be used as a tissue specific biomarker to determine the likelihood of organ failure due to allograft rejection. DdcfDNA has also been utilized in the risk stratification of organ rejection post heart transplant in pediatrics and adults as reported by North et al. (40).

While these studies highlight the potential of cfDNA as a novel biomarker for organ dysfunction, further work is required to identify tissue-specific DNA which would more reliably diagnose organ failure in children.

## Micro RNA

Micro RNAs (miRNA) are small, single-stranded, non-coding ribonucleotides that negatively regulate gene expression at the post-transcriptional level and play a role in cell proliferation, differentiation, repair and apoptosis (41). While each miRNA may regulate hundreds of genes, multiple miRNAs may work cooperatively to target one gene and despite their vast heterogeneity, miRNAs can display tissue-specific expression patterns (41, 42). MiRNAs are actively released into circulation as well as extracellular spaces including saliva and urine in response to toxic cellular insults either as extracellular vesicles or bound to RNA-binding proteins. This protein bound complex allows miRNA to persist in a stable form that is resistant to degradation, making them ideal biomarker candidates.

While the prognostic value of miRNAs has been reported for the prediction of outcomes for oncology patients as well as the prediction of multiple types of organ failure, there is a scarcity of data on its use to predict organ failure in pediatric patients (43, 44). Schneider et al showed that increased levels of miRNA-21, miRNA-126 and miRNA-423 predicted more favorable outcomes for patients with heart failure with fewer hospital readmissions (45). Cakmak et al demonstrated upregulation of miRNA-182 in patients with heart failure and found that it was superior to pro-brain type natriuretic peptide (pro-BNP), a biomarker often used in predicting heart failure. This study also showed that multiple miRNAs can undergo significant dysregulation in patients with organ failure and isolated 28 dysregulated miRNAs in patients with heart failure (20). In an attempt to increase its prognostic significance, Goren et al showed that multiple miRNA values can be combined into a single “miRNA score” for heart failure (46). Similarly, Zhu et al showed the benefit of combining multiple miRNAs with an already established grading system for the prediction of acute respiratory distress syndrome (21).

MiRNA profiling has also been shown to be useful in distinguishing the etiology of organ failure. Patients with acute liver failure (ALF) as a result of either HCV or cirrhosis were both found to have elevated levels of miRNA122. (47, 48). Weis et al also highlighted its potential in differentiating hepatocellular carcinoma (HCC) from liver dysfunction secondary to cirrhosis without the need for a biopsy and showed it was superior to alpha fetal protein, the only currently used biomarker utilized for HCC (49). Additionally, serum miRNAs have been shown to be a predictive biomarker in AKI. Aguado-Fraile et al verified that elevated serum miRNA-26b-5p, miRNA-27a-3p,miRNA-93-3p and miRNA-127-3p levels in patients with normal kidney function prior to cardiac surgery predicted AKI development post-operatively. Furthermore, this change in miRNA levels occurred days before the serum creatinine increased, highlighting both its worth as a novel predictor of AKI and the correlation of miRNA level with disease severity (29).

While the use of miRNA as a biomarker for organ failure is promising, further work is needed to determine the prognostic significance of these abnormal miRNAs in order to maximize their clinical utility in pediatric organ failure.

## Endothelial Progenitor Cells

Endothelial progenitor cells (EPCs) are a subpopulation of stem cells that have been identified in the peripheral blood in response to endothelial damage secondary to insults, including vascular injury and ischemia. They are believed to be critical to maintain vascular integrity and endogenously restore and repair damaged vascular endothelium. Although EPCs have been studied as biomarkers for organ dysfunction, current studies in regards to EPCs and their role in cancer focus on their role as prognostic biomarkers (50).

Increased levels of EPCs are associated with decreased cardiovascular mortality amongst patients with coronary artery disease (51). Moazzami et al also showed that decreased levels of a subpopulation of EPCs known as circulating progenitor cells (CPCs) are associated with a worse prognosis and is a stronger factor in outcomes than the presence of stress-induced myocardial ischemia in patients with coronary artery disease (52). More recently, Lieu et al demonstrated the use of EPCs in predicting persistent organ failure in patients with severe acute pancreatitis (SAP) and showed the level of EPCs correlate negatively to clinical scores that are used to grade SAP including the Ranson and acute physiology and chronic health evaluation (APACHE) scores (53). Interestingly, patients with persistent organ failure had lower levels of EPCs, though still elevated from the healthy volunteers than those with transient organ failure. Tapia et al showed that though there was an increase in the number of EPCs, the protein expression of endothelial growth factor receptor-2 (VEGFR-2) and CD34 in the CEPCs (CD133+) was lower in critically ill patients with septic or non-septic shock and lowest in patients that did not survive (35).

In a pediatric study, EPCs were reported to be highly correlated with pulmonary arterial hypertension in pediatric patients with congenital heart disease (54). EPCs have also been reported to stratify risk in adult patients with chronic kidney disease (CKD),however the same did not hold true for pediatric patients with predialysis CKD (55).

While the role of EPCs as a biomarker for organ failure is promising, these cell levels are altered by multiple other factors including age, underlying chronic diseases such as diabetes mellitus, frequency of dialysis and the use of immunosuppressive drugs thus limiting its current value and application to pediatrics (56).

## Exosomes

Exosomes are micro vesicles, roughly 30-100 nm in diameter, released from cells after fusion with an intermediate endocytic compartment (57). These microvesicles contain various biomolecular cargo that are transported across cell membranes. Under physiological conditions, exosomes are present and widely distributed in bodily fluids and are often treated as therapeutic targets or diagnostic and prognostic biomarkers in patients with cancer. Their widespread availability also make them an easily accessible noninvasive potential biomarker (58–60).

Sole et al examined miR-29c in urinary exosomes to determine whether patients with lupus nephritis would develop end-stage renal disease. They found that the contents of these microvesicles correlated with renal chronicity but not with renal function, suggesting that it could possibly be used as an early biomarker for development of renal fibrosis (31). Exosomes have also been used as biomarkers of renal injury within the context of preeclampsia. Gilani et al found that in women with preeclamptic podocyte related renal injuries, a significantly higher ratio of podocin-positive to nephrin-positive extracellular vesicles was found when compared to the urine of women with normotensive pregnancies, indicating proteinuria and renal injury (61).

Njock et al analyzed exosomes in the sputum to find biomarkers for idiopathic pulmonary fibrosis. They found exosomes containing mir-142-3p were negatively correlated with the diffusing capacity of the lungs and therefore severity of the disease (22). Lambrecht et al investigated the role of plasma exosomal miRNAs in distinguishing between healthy individuals and patients with hepatitis B and C who had early stage fibrosis. They found that certain exosomal miRNA cargos could be used to distinguish between healthy patients and HBV and HCV groups. However, whether these exosomes can diagnose liver fibrosis of any origin remains unclear (27). While the aforementioned studies highlight the potential for exosomes as a biomarker of organ failure in adults, there is currently a lack of similar research in pediatrics and further research is needed to evaluate its applicability in children.

## Nucleosomes

Nucleosomes are complexes composed of a histone core surrounded by DNA base pairs. Under normal physiological conditions, nucleosomes are released during apoptosis. However in pathological conditions, as in the case of organ failure, the normal phagocytic clearance mechanisms are overwhelmed and nucleosomes are released into circulation (62). These nucleosomes are remarkably stable and can be measured from the serum using immunoassay techniques. Current studies focus on their role as biomarkers with which to diagnose patients with cancers or with which to stratify patients for more tailored therapy (63).

Nucleosome levels have also been shown to be capable of differentiating the acuity and pathogenesis of organ failure. In a cohort of surgical and non-surgical ICU patients, patients who were septic had higher levels of nucleosomes upon admission than those who were not. This, however, was comparable to already established clinical scoring systems and did not provide additional predictive benefit (64). Craig et al showed that there are elevated nucleosome levels in patients with ALF in comparison to patients with chronic liver disease. However, there was no significant difference in nucleosome levels in severity of liver dysfunction or outcome (65). Additionally, nucleosome levels have been shown to be adversely related to cardiac outcomes and also elevated in familial cardiomyopathy (66, 67) In pediatrics, elevated levels of nucleosomes were found to be associated with increased mortality in pediatric acute respiratory distress syndrome (p<0.001) (68).

## Circulating Mitochondrial DNA

Mitochondria are cellular organelles whose DNA (mtDNA) has been found to reflect a potential damage-associated molecular pattern. They are released extracellularly during necrosis in response to inflammation and have been found to be associated with the severity of a variety of diseases. Thus, mtDNA may be a potential biomarker for organ failure (69, 70). Publications that have examined the role of mtDNA in cancer focus on both copy number as a diagnostic and prognostic marker and on mutations within mtDNA as an additional prognostic marker (71–74).

Timmermans et al found mtDNA levels to be elevated in patients with septic shock. Di Caro et al additionally demonstrated this facet in pediatric patients (75). Dhondup showed low circulating levels of mtDNA to be associated with greater mortality in chronic heart failure. McGill et al found that elevated levels of mtDNA are associated with poorer outcomes in patients with liver failure (76–78). MtDNA can be isolated from the blood as well as urine and the latter has been explored as a biomarker for kidney injury. Eirin et al demonstrated elevated levels of mtDNA in patients with hypertension and its correlation with renal dysfunction, while Hu et al demonstrated urinary mtDNA to be a more sensitive predictor of AKI in post-operative patients than serum creatinine (32, 79). MtDNAs have also been shown to play a role in organ dysfunction and rejection post-transplant. Pollara et al demonstrated a correlation between elevated donor plasma mitochondrial DNA levels and early allograft dysfunction in liver transplant recipients, suggesting a role for circulating mtDAMPs in allograft outcomes (80).

## Histones

Histones are proteins found extracellularly and within the nucleus that have been found to be elevated in patients with cancer and are associated with cell death and toxicity (81, 82). Abrams et al examined circulating histone levels in patients with trauma-induced lung injuries and found that high levels of circulating histones were associated with the incidence of acute lung injury and Sequential Organ Failure Assessment (SOFA) scores (83). Liu et al demonstrated that histones predicted disease severity in severe acute pancreatitis with higher predictive values for persistent organ failure and mortality than the currently used scoring indices for pancreatitis (34). Elevated histone levels have also been shown in patients with acute liver injury, acute kidney damage, and correlate with markers of disease severity and mortality (84, 85).

Other studies have analyzed the relationship between high levels of circulating histones and multiple organ failure. In patients with multiple organ dysfunction syndrome, there was a strong correlation between circulating histone levels and markers of organ injury, disease severity and p-SOFA scores. Furthermore, the authors observed that sera from patients with high histone levels were non-selectively toxic to primary cells from a variety of organs, including the lungs, livers and kidneys and therefore a possible mediator for multiple organ dysfunction syndrome (86).

## Discussion

Biomarkers are surrogate indirect indicators of a pathological or physiological process. In pediatrics, there has been limited progress in the identification and development of biomarkers to predict organ failure, its severity and outcome and its response to therapy. Identifying suitable biomarkers for organ dysfunction in pediatrics could potentially help improve risk stratification and possibly patient outcomes. It may also help to reduce the psychological stress, patients and their families may experience for more invasive procedures as well as reduce the health care costs and risks associated with these procedures which often require sedation.

Currently for biomarkers reported in the literature, there is a high degree of heterogeneity in the levels amongst patients based on age, sex and existing comorbidities. Levels of cfDNA for example are much higher in elderly patients due to decreased level of clearance by phagocytosis whilst changes in cfDNA methylation occur rapidly during childhood (87, 88). Limitations of these reported studies include lack of age-matched controls, limited sample sizes and lack of studies that included pediatric patients. These limitations make it impossible to assess age-related changes of the biomarkers reported and limits its generalizability to pediatric patients where age and weight specific normal ranges is unknown. Barriers to development of pediatric biomarkers include low prevalence of disease in pediatric vs adult patients and parental reluctance to participate in research, particularly for healthy controls making sample acquisition challenging (89).

Additionally in order to develop meaningful and clinically useful biomarkers, further research is needed to establish the optimal timing and method of sample collection as well as standardization of laboratory techniques. It should also be noted that none of these potential biomarkers in this review are truly organ-specific and may be a marker for multiple diseases thereby limiting its usefulness. Further work is therefore needed in distinguishing each biomarker from their respective molecular or cellular background in order to enhance its clinical utility. Presently there are multiple ongoing pediatric clinical trials to identify biomarkers in pediatric patients with lung, cardiac and renal injury amongst others (90–95).

Current ongoing or planned trials for pediatric oncology patients include the identification of biomarkers to detect cardiac injury and renal injury post chemotherapy (96–99).

Although there is still much work to be done, among pediatric oncology patients, there is promising utility for several of the biomarkers reviewed. Future studies might examine (i) the use of cfDNA and mtDNA post hematological or solid organ transplant in detecting organ rejection (ii) the use of MiRNA to determine risk of AKI among patients with post-surgical tumor resection (iii) serial sampling of EPCs in predicting progressive organ failure (iv) the use of histones as a predictor for multi-organ dysfunction syndrome and (v) the use of exosomes and nucleosomes to predict persistent or chronic organ failure in pediatric cancer patients.

## Conclusions

Although many potential biomarkers exist to predict organ failure among pediatric cancer patients, all come with shared and unique challenges that limit their clinical value at this time. Additionally, while much research has been focused on heart failure and sepsis, a leading cause of multi-organ failure, there has been little headway in the application of these potential biomarkers to lung injury, for which there is currently no prognostic score or standard severity grading and diagnosis remains reliant on clinical markers. Despite the need for further studies, the data is promising. Pediatric oncology and critical care investigators should be encouraged to investigate these biomarkers further as they hold great potential for future use.

## Author Contributions

LD, SY, and DR were all major contributors in writing and editing this manuscript. RS, DP, KM, and SL were major contributors in editing the manuscript. All authors contributed to the article and approved the submitted version.

## Conflict of Interest

The authors declare that the research was conducted in the absence of any commercial or financial relationships that could be construed as a potential conflict of interest.
